# Anabolic–Androgenic Steroid Use, All-Cause Mortality, and Major Cardiovascular Outcomes: A Systematic Review and Meta-Analysis

**DOI:** 10.3390/medsci14060678

**Published:** 2026-10-15

**Authors:** María Lourdes Del Río-Solá, Juan Martin-Hernandez, Alejandro Santos-Lozano

**Affiliations:** 1 Department of Surgery, Ophthalmology, Otorhinolaryngology and Physiotherapy, University of Valladolid, Ramón y Cajal Ave, No. 7, 47005 Valladolid, Spain; 2 Department of Vascular Surgery, University Clinical Hospital of Valladolid, Ramón y Cajal Ave, No. 3, 47003 Valladolid, Spain; 3 Department of Health Sciences, Miguel de Cervantes European University, Padre Julio Chevalier St., No. 2, 47012 Valladolid, Spain

**Keywords:** anabolic-androgenic steroids, mortality, cardiovascular disease, cardiomyopathy, global longitudinal strain, meta-analysis

## Abstract

**Background/Objectives:** Non-medical anabolic-androgenic steroid (AAS) use has been associated with adverse cardiac remodelling, but the magnitude of excess all-cause mortality and the cardiac phenotype of former users remain incompletely defined. **Methods:** We systematically reviewed studies reporting mortality, major cardiovascular outcomes, or cardiac function after cessation in AAS-exposed individuals. All-cause mortality was pooled from comparative cohorts with individually ascertained exposure using random-effects models; global longitudinal strain (GLS) and left ventricular ejection fraction in former users were pooled as mean differences. Risk of bias was assessed with ROBINS-E and certainty with GRADE. **Results:** Fifteen primary reports were included. Across three cohorts, AAS exposure was associated with higher all-cause mortality (risk ratio 2.57, 95% CI 1.98–3.33; I^2^ = 0%; 95% prediction interval 0.47–13.87), with consistent sensitivity analyses. In a single large matched cohort, adjusted hazard ratios for cardiovascular outcomes ranged from 2.26 to 8.90, highest for cardiomyopathy. Former users had worse GLS than AAS-naïve controls (3 studies; mean difference +0.90 percentage points, 95% CI +0.13 to +1.66), whereas the pooled ejection-fraction estimate was exploratory and highly heterogeneous. **Conclusions:** AAS exposure was associated with higher all-cause mortality in three observational cohorts, but residual confounding, differing follow-up, and the small evidence base preclude causal inference. Cardiovascular associations from a single cohort require independent replication, and evidence on residual myocardial dysfunction in former users is of very low certainty.

## 1. Introduction

Anabolic–androgenic steroids (AAS) are synthetic derivatives of testosterone used therapeutically in selected conditions but also consumed non-medically to increase skeletal muscle mass, strength, and physical appearance. Non-medical use extends from elite sport to recreational resistance training and bodybuilding, where exposure may be supraphysiological, prolonged, cyclical, and combined with other performance-enhancing substances. The European Association of Preventive Cardiology has identified AAS among doping substances with potentially serious cardiovascular consequences [1].

A growing imaging literature describes a characteristic cardiovascular phenotype in long-term users, including increased left ventricular (LV) mass and wall thickness, impaired LV ejection fraction (LVEF), reduced global longitudinal strain (GLS), higher blood pressure, adverse lipid profiles, and, in some cohorts, premature coronary atherosclerosis [2,3,4,5].

The clinical relevance of this phenotype has become clearer with contemporary registry and forensic data. A large Danish matched cohort reported increased risks of myocardial infarction, coronary revascularization, venous thromboembolism, arrhythmia, cardiomyopathy, and heart failure among AAS users [6], while forensic and pathological studies provide complementary biological evidence: cardiomegaly, LV hypertrophy, myocardial fibrosis, necrosis, myocyte disarray, coronary atherosclerosis, and thrombosis have been described in AAS-associated deaths, frequently in the setting of polypharmacy [7,8,9,10].

However, the evidence base is methodologically heterogeneous. Some cohorts use analytically confirmed exposure, whereas historical power-sport cohorts infer AAS use from the sporting era and discipline; forensic series often lack a population denominator; and bodybuilding cohorts do not systematically verify individual exposure [11,12,13,14,15]. Importantly, recent population-based evidence has demonstrated increased mortality among AAS users [15], strengthening the association between AAS exposure and all-cause mortality but leaving unresolved whether this mortality signal is consistent across independent cohorts with individually ascertained exposure.

Previous reviews have summarized cardiovascular toxicity, and a recent meta-analysis quantified structural and functional echocardiographic abnormalities in AAS users [2,7,10]. However, all-cause mortality has not been quantitatively synthesized across comparative cohorts with individually ascertained AAS exposure, and its relationship with the broader spectrum of major cardiovascular outcomes and cardiac function in former users remains incompletely characterized. Establishing the magnitude and consistency of these associations is clinically relevant because evidence from individual cohorts cannot determine whether the observed mortality signal is reproducible across independent populations.

We therefore aimed to (i) quantify all-cause mortality across comparative cohorts with confirmed AAS exposure; (ii) characterize major cardiovascular outcomes without pooling biologically distinct endpoints; (iii) quantify residual global longitudinal strain and left ventricular ejection fraction differences in former AAS users compared with AAS-naïve controls; and (iv) integrate mechanistic, forensic, and high-risk sport evidence to identify pathways and effect modifiers relevant to cardiovascular risk.

## 2. Methods

### 2.1. Protocol and Reporting

The review was conducted in accordance with the Preferred Reporting Items for Systematic Reviews and Meta-Analyses (PRISMA 2020) statement and the Meta-analysis Of Observational Studies in Epidemiology (MOOSE) recommendations [16,17]. The review protocol was prospectively registered in PROSPERO (CRD420261518294; https://www.crd.york.ac.uk/prospero/display_record.php?ID=CRD420261518294 (accessed on 13 October 2026)). No amendments were made to the registered protocol during the conduct of the review; the additional sensitivity analyses described in Section 2.7 were added post hoc in response to peer review and are identified as such.

### 2.2. Eligibility Criteria

Eligible reports evaluated non-medical AAS exposure in human populations and reported at least one prespecified domain: all-cause or cause-specific mortality; sudden cardiac death; major cardiovascular outcomes; cardiac structure or function relevant to former users after cessation; or pathological/forensic evidence directly informing cardiovascular mechanisms. Comparative cohorts, matched registry studies, longitudinal or cross-sectional imaging studies, and selected forensic or pharmacovigilance studies were retained for the relevant qualitative domains.

For the primary mortality meta-analysis, eligibility was deliberately stricter: exposure had to be individually confirmed by analytical testing or a direct anti-doping ascertainment process, an eligible comparator had to be present, and event counts had to permit calculation of a common relative measure. Historical power-sport cohorts with probable but unconfirmed individual exposure were excluded from the primary pooled estimate.

For the post-cessation cardiac-function meta-analysis, studies had to report a former/discontinued AAS group and an AAS-naïve comparator with extractable global longitudinal strain and/or left ventricular ejection fraction data. These analyses compare groups at follow-up and therefore estimate residual between-group differences after cessation rather than individual recovery trajectories. Short off-cycle interruptions were not considered equivalent to established cessation in the primary analysis.

### 2.3. Information Sources and Search Strategy

A comprehensive search was conducted across MEDLINE/PubMed, Embase, Scopus, and Web of Science from database inception and updated through August 2026. Search concepts combined terms for anabolic-androgenic steroids, androgen misuse, bodybuilding/doping, mortality, sudden death, myocardial infarction, coronary disease, thrombosis, arrhythmia, cardiomyopathy, heart failure, echocardiography, ventricular function, and strain. Reference lists of eligible studies and relevant systematic reviews were hand-searched. Database-specific search strategies are reported in the Supplementary Methods.

**Figure 1:**
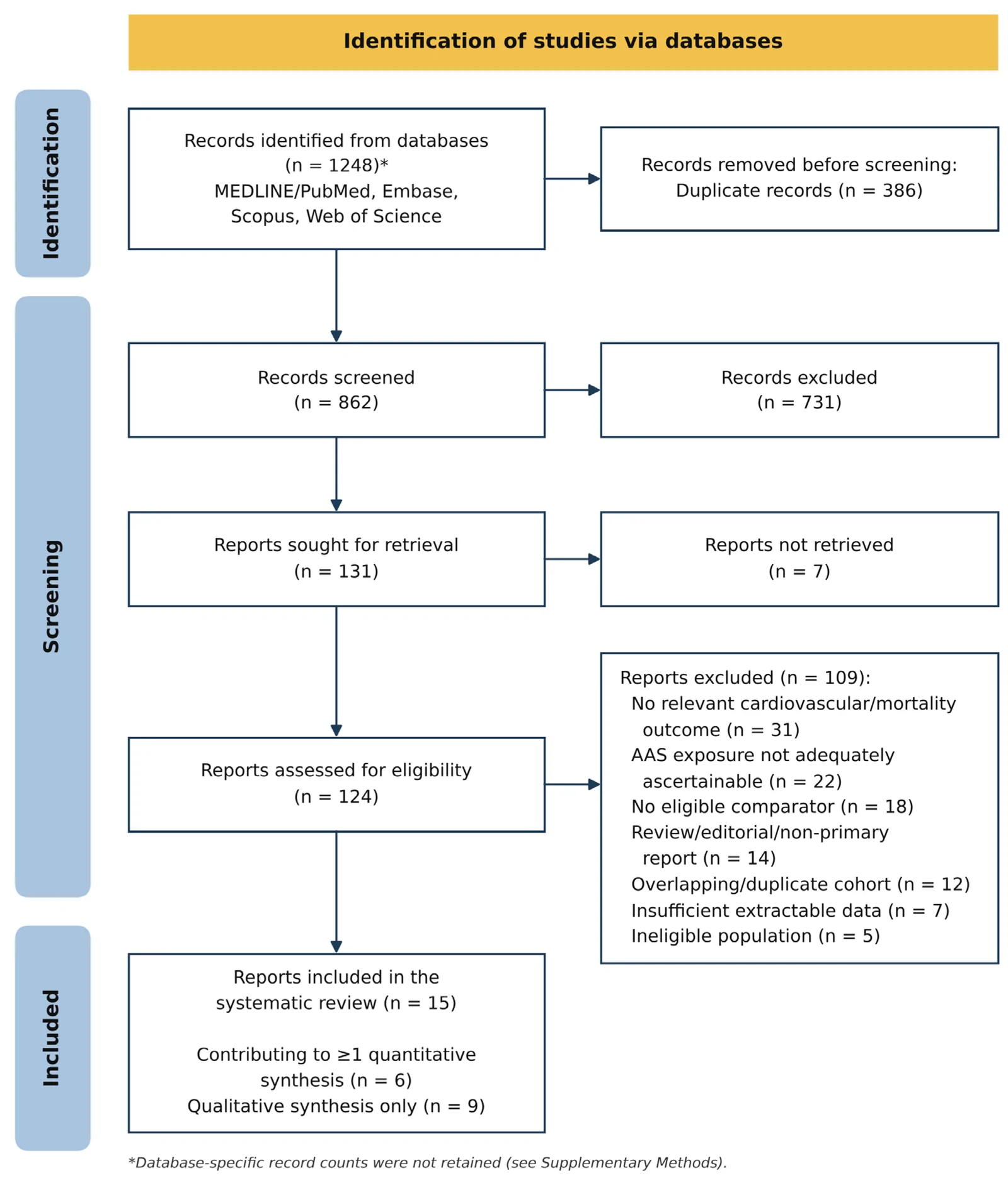
PRISMA 2020 flow diagram of study selection. Fifteen primary reports were included in the systematic review, of which six contributed data to at least one quantitative synthesis. Full-text exclusion reasons are shown explicitly; secondary reviews and other non-primary publications used only for contextual interpretation or citation tracking were not counted as included primary studies. Database-specific record counts were not retained (see Supplementary Methods).

The study selection process is summarized in the PRISMA flow diagram (Figure 1).

### 2.4. Study Selection and Data Extraction

Titles and abstracts were screened independently by two reviewers against prespecified criteria, followed by independent full-text eligibility assessment by the same reviewers; disagreements at either stage were resolved by discussion or, if unresolved, by adjudication from a third reviewer. Data were extracted independently in duplicate using a standardized extraction form and cross-checked for accuracy, with discrepancies resolved by consensus. Extracted variables included country, study design, participant characteristics, exposure definition, comparator, follow-up, mortality and cardiovascular event counts, adjusted effect estimates, echocardiographic outcomes, AAS duration, cessation status, concomitant substances, and information required to identify cohort overlap.

Potentially overlapping publications were mapped before quantitative synthesis. For each outcome, the largest and most recent non-overlapping dataset was preferred. The 2019 Danish study was therefore not counted as an independent mortality cohort when the expanded 2024 Danish mortality analysis was available; the 2025 Circulation report from the same population was retained only for distinct cardiovascular outcomes.

### 2.5. Outcomes

The primary outcome was all-cause mortality among participants with confirmed AAS exposure. Secondary clinical outcomes were prespecified as acute myocardial infarction, coronary revascularization, venous thromboembolism, ischaemic stroke, arrhythmia, cardiomyopathy, heart failure, and cardiac arrest. In this review, the umbrella term “major cardiovascular outcomes” refers to this heterogeneous set of clinically important endpoints and is not intended to denote a conventional composite major adverse cardiovascular event endpoint. Cause-specific natural and unnatural mortality were summarized when directly reported. Cardiac function in former users was evaluated using global longitudinal strain and left ventricular ejection fraction; because all eligible studies reported these outcomes in common units, mean difference rather than standardized mean difference was selected for the primary analysis.

### 2.6. Risk of Bias and Certainty of Evidence

Risk of bias in non-randomized follow-up studies of exposure effects contributing to the mortality and post-cessation imaging evidence was assessed using ROBINS-E (version 24 March 2024), addressing bias due to confounding, measurement of exposure, selection of participants, post-exposure interventions, missing data, outcome measurement, and selection of the reported result [18]. Risk-of-bias judgements were made independently by two reviewers for each contributing study, with disagreements resolved by discussion or, if unresolved, adjudication by a third reviewer. Study-level, domain-specific judgements are provided in Supplementary Table S5. Certainty of evidence was rated by outcome using the GRADE framework, considering risk of bias, inconsistency, indirectness, imprecision, publication bias, and potential upgrading factors [19,20].

### 2.7. Statistical Analysis

For mortality, raw binary data were synthesized as risk ratios (RRs) with 95% confidence intervals (CIs) using an inverse-variance random-effects model, with between-study variance estimated by restricted maximum likelihood (REML), the default estimator in SPSS. This approach was selected because directly comparable adjusted time-to-event estimates were not available for all eligible cohorts. Hazard ratios (HRs), when available, were retained in the study-level tables and emphasized in interpretation as supportive estimates that account for follow-up time and, when adjusted, for measured confounding. Because event-based RRs do not account for differences in follow-up duration, available absolute mortality incidence rates per 1000 person-years were also extracted or calculated from reported person-time when possible.

Continuous cardiac outcomes were pooled as mean differences (MDs) with 95% CIs. Where studies reported standard errors (SEs), these were converted to standard deviations (SDs) using SD = SE × √n. Where follow-up values were reported as medians and interquartile ranges, approximate means and SDs were derived using the method of Wan et al. [21], based on the number of participants with available measurements for each outcome, to permit quantitative synthesis. All transformations are explicitly identified in the study-level extraction table.

Statistical heterogeneity was assessed using Cochran’s Q test, the I^2^ statistic, and between-study variance (τ^2^). For the primary mortality synthesis, a 95% prediction interval was calculated using the Higgins-Thompson-Spiegelhalter approach with a t distribution and k-2 degrees of freedom. The leave-one-out analysis was prespecified; a fixed-effect model, the 95% prediction interval, a restricted analysis limited to the two cohorts with analytically confirmed AAS exposure, and an analysis excluding cohorts of uncertain independence were added post hoc in response to peer review. Because I^2^ was derived from the REML estimate of τ^2^, it can be non-zero when Cochran’s Q is smaller than its degrees of freedom. Because fewer than 10 studies contributed to each pooled analysis, funnel plots and formal statistical tests for small-study effects were not performed. Formal meta-regression was not undertaken for the same reason; possible sources of between-study heterogeneity, including exposure ascertainment, comparator population, follow-up duration, and time since cessation, were explored narratively. Major cardiovascular outcomes from the large Danish matched cohort were summarized using the reported adjusted HRs and 95% CIs without formal pooling because the endpoints were clinically distinct and originated from the same source cohort.

For the primary mortality meta-analysis, leave-one-out sensitivity analysis was performed by sequentially excluding each cohort to determine whether the pooled association was disproportionately influenced by any single study. The fixed-effect analysis assessed sensitivity to the random-effects specification, while the ascertainment sensitivity analysis excluded the Danish anti-doping cohort and retained only analytically confirmed AAS-positive cohorts, and the independence sensitivity analysis excluded Petersson et al. because its participants could not be confirmed to be distinct from those of Thiblin et al.

Statistical analyses were performed using IBM SPSS Statistics, version 29.0 (IBM Corp., Armonk, NY, USA). Additional data processing, independent verification of effect estimates, sensitivity analyses, and graphical visualization were performed using Python (version 3.15.0; Python Software Foundation, Wilmington, DE, USA), with NumPy (version 2.5.4), pandas (version 3.0.6), SciPy (version 1.18.1), statsmodels (version 0.15.0), and Matplotlib (version 3.11.2) used as applicable.

## 3. Results

### 3.1. Study Selection

Fifteen primary reports were included in the systematic evidence synthesis, spanning national registry cohorts, medically referred AAS-testing cohorts, cardiac imaging cohorts, forensic investigations, pharmacovigilance data, and competitive bodybuilding/power-sport populations (Figure 1). Six unique reports contributed to at least one mortality or post-cessation cardiac-function quantitative synthesis; study characteristics and their roles in the evidence synthesis are summarized in Table 1.

### 3.2. Exposure Certainty and Cohort Overlap

Exposure ascertainment varied substantially across the included studies. Petersson et al. and Thiblin et al. used analytically confirmed AAS exposure [12,13], whereas the Danish studies identified men who received an AAS-related anti-doping sanction through a national fitness-centre testing programme [6,14,15]. In the Danish programme, an AAS-related sanction could follow either a positive test or refusal to provide a urine sample; in this source population, 644 of the 1189 sanctioned men had refused testing and 545 had tested positive [14], so exposure in approximately half of the Danish cohort was not analytically confirmed, and this cohort was evaluated separately in an ascertainment sensitivity analysis. Historical Finnish and Swedish power-sport cohorts had probable exposure at the cohort level but no individual verification and were therefore retained as contextual evidence rather than included in the primary confirmed-exposure mortality meta-analysis [11,22]. The 2019, 2024, and 2025 Danish reports represented sequential analyses of the same anti-doping source population; only the most informative report for each outcome was used to avoid double counting [6,14,15]. Likewise, the 2025 Fyksen study was a long-term follow-up of the source cohort reported in 2022 and contributed only the relevant follow-up outcomes [3,23]. No sequential reports from the same source population contributed simultaneously to the primary mortality meta-analysis. The two Swedish cohorts (Petersson et al. and Thiblin et al.) were both drawn from individuals tested at the doping laboratory of Huddinge University Hospital, the only Swedish laboratory performing AAS analysis for most of the study periods, but from largely consecutive testing periods (1995–2001 and mainly 2002–2009, respectively). Because Thiblin et al. also included 102 men (57 AAS-positive) with earlier tests of unknown date, partial overlap could not be excluded, and a sensitivity analysis excluding Petersson et al. was performed (Section 3.3). Detailed overlap rules are provided in Supplementary Table S1.

### 3.3. Primary Meta-Analysis: All-Cause Mortality

**Figure 2:**
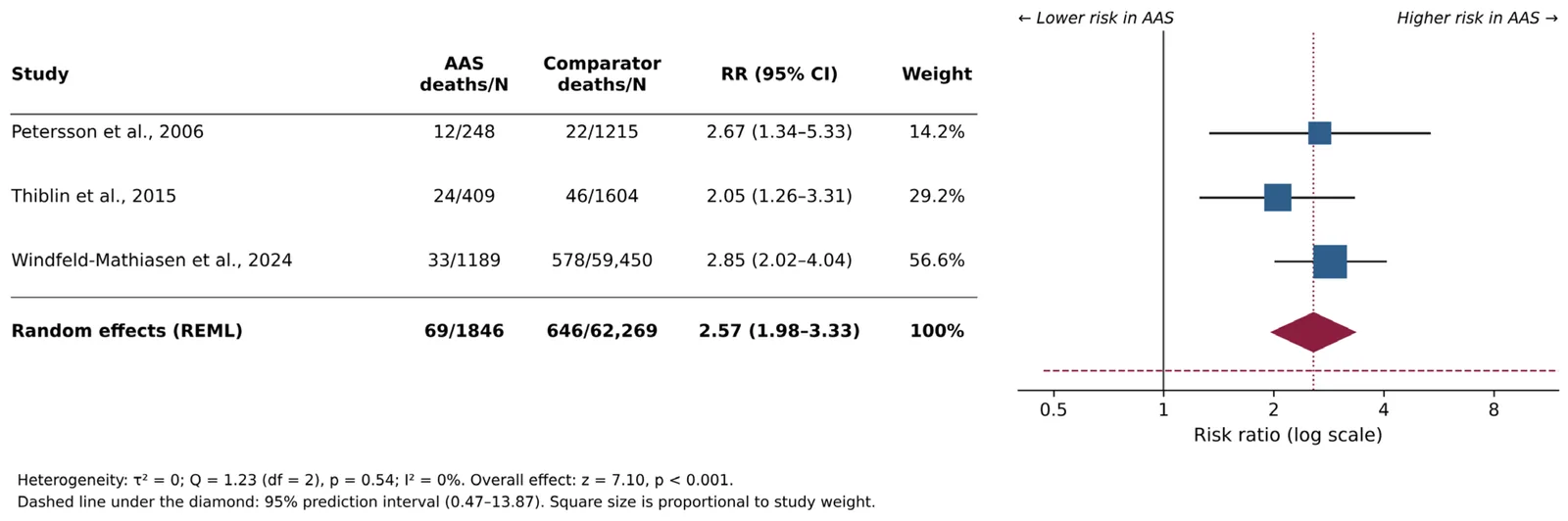
Meta-analysis of all-cause mortality in comparative cohorts with confirmed anabolic-androgenic steroid exposure. Forest plot showing study-specific and pooled risk ratios (RRs) with 95% confidence intervals (CIs) for all-cause mortality among individuals with individually ascertained anabolic-androgenic steroid (AAS) exposure compared with non-exposed comparators. Three independent cohorts contributed to the quantitative synthesis. The random-effects pooled RR was 2.57 (95% CI 1.98–3.33), with no observed between-study heterogeneity (I^2^ = 0%). Squares represent study-specific estimates, with their size proportional to study weight; horizontal lines indicate 95% CIs, and the diamond represents the pooled estimate; the dashed line below the diamond indicates the 95% prediction interval (0.47–13.87), and the x-axis is on a logarithmic scale.

Three comparative cohorts with individually ascertained AAS exposure contributed to the primary all-cause mortality meta-analysis: Petersson et al., Thiblin et al., and Windfeld-Mathiasen et al. [12,13,15]. AAS exposure was associated with a significantly increased risk of all-cause mortality (RR 2.57, 95% CI 1.98–3.33; *p* < 0.001), based on 69 deaths among 1846 AAS-exposed participants and 646 deaths among 62,269 comparators (Figure 2). Observed statistical heterogeneity was low (Q = 1.23, *p* = 0.54; I^2^ = 0%; τ^2^ = 0), but the 95% prediction interval was wide (0.47–13.87) because only three cohorts were available and should not be interpreted as evidence that the underlying populations were clinically homogeneous. The fixed-effect sensitivity analysis produced the same pooled estimate (RR 2.57, 95% CI 1.98–3.33). In post hoc sensitivity analyses, restricting the synthesis to the two cohorts with analytically confirmed AAS exposure (equivalent to the leave-one-out analysis omitting the Danish cohort) yielded RR 2.23 (95% CI 1.50–3.31), and excluding Petersson et al., whose independence from Thiblin et al. could not be verified, yielded RR 2.53 (95% CI 1.84–3.46). Leave-one-out analyses remained statistically significant irrespective of which cohort was excluded (RR range 2.23–2.82). Time-to-event estimates, where available, were directionally consistent: Thiblin et al. reported an adjusted HR of 1.69 (95% CI 1.02–2.82) after adjustment for education, country of birth, and previous alcohol and drug abuse, whereas Windfeld-Mathiasen et al. reported an age-matched but otherwise unadjusted HR of 2.81 (95% CI 1.98–3.99). The only confounder-adjusted estimate (Thiblin et al.) was smaller than the corresponding crude RR (2.05), indicating that part of the crude association may reflect measured confounding. Absolute mortality rates were 14.0 versus 6.7 deaths per 1000 person-years in Thiblin et al. and 2.48 versus 0.88 deaths per 1000 person-years in the Danish cohort; person-time was not reported in a form permitting a comparable incidence-rate calculation for Petersson et al. GRADE certainty for the pooled mortality outcome remained low because all evidence was observational and vulnerable to residual confounding and selection effects despite the consistency of effect direction and sensitivity analyses (Table 2).

### 3.4. Major Cardiovascular Outcomes

**Figure 3:**
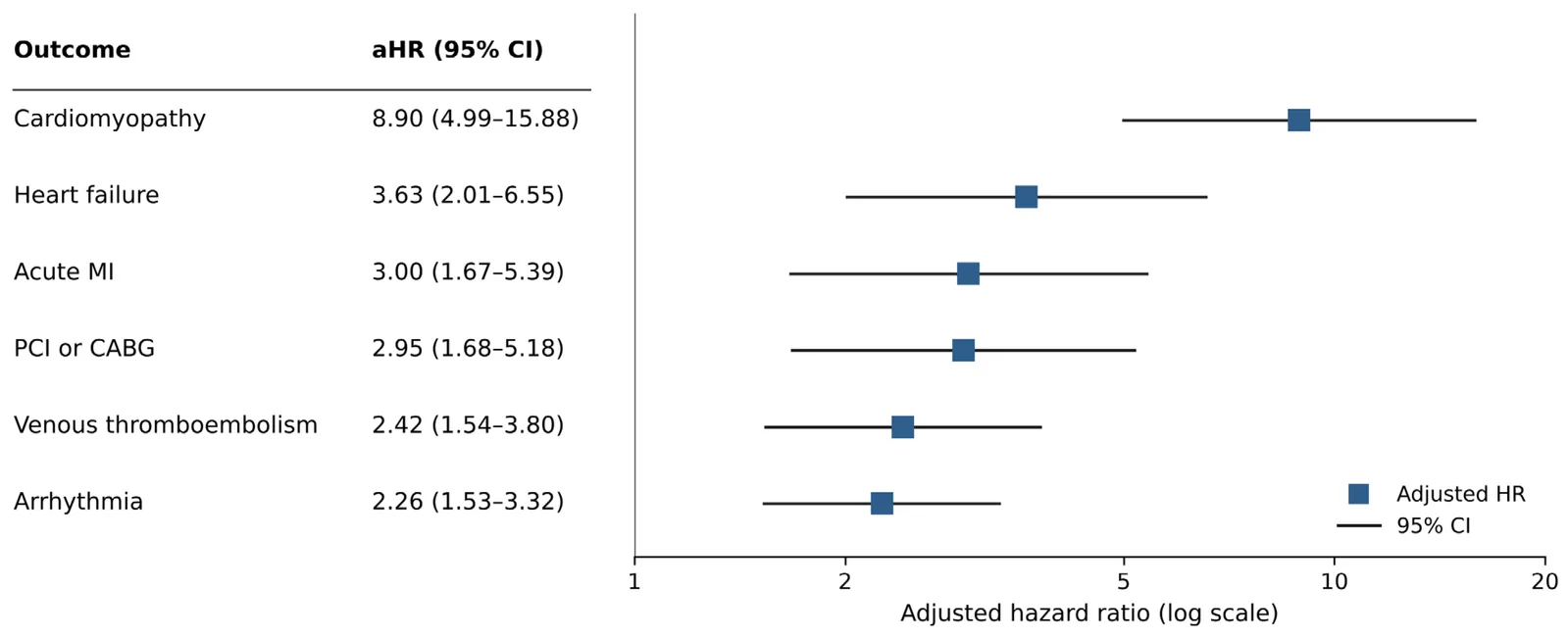
Major cardiovascular outcomes associated with anabolic-androgenic steroid exposure in the Danish matched cohort. Adjusted hazard ratios (HRs) with 95% confidence intervals (CIs) for major cardiovascular outcomes among individuals with anabolic-androgenic steroid (AAS) exposure compared with matched population controls in the Danish cohort. Outcomes include acute myocardial infarction, coronary revascularization, venous thromboembolism, arrhythmia, cardiomyopathy, and heart failure. Adjusted HRs ranged from 2.26 to 8.90 across the reported outcomes. No pooled estimate was calculated because these represent clinically distinct endpoints derived from the same source cohort.

The largest contemporary analysis of incident cardiovascular disease included 1189 AAS users and 59,450 matched controls followed for approximately 11 years [6]. In this single Danish source cohort, AAS exposure was associated with cardiomyopathy (aHR 8.90, 95% CI 4.99–15.88), heart failure (aHR 3.63, 95% CI 2.01–6.55), acute myocardial infarction (aHR 3.00, 95% CI 1.67–5.39), PCI or CABG (aHR 2.95, 95% CI 1.68–5.18), venous thromboembolism (aHR 2.42, 95% CI 1.54–3.80), and arrhythmia (aHR 2.26, 95% CI 1.53–3.32) [6]. Ischaemic stroke and cardiac arrest could not be statistically reported because fewer than five exposed events occurred. Because these estimates derive from one matched cohort, they should be interpreted as strong but not yet independently replicated associations rather than as pooled evidence across multiple populations (Figure 3; Supplementary Table S3).

### 3.5. Cardiac Function After AAS Cessation

**Figure 4:**
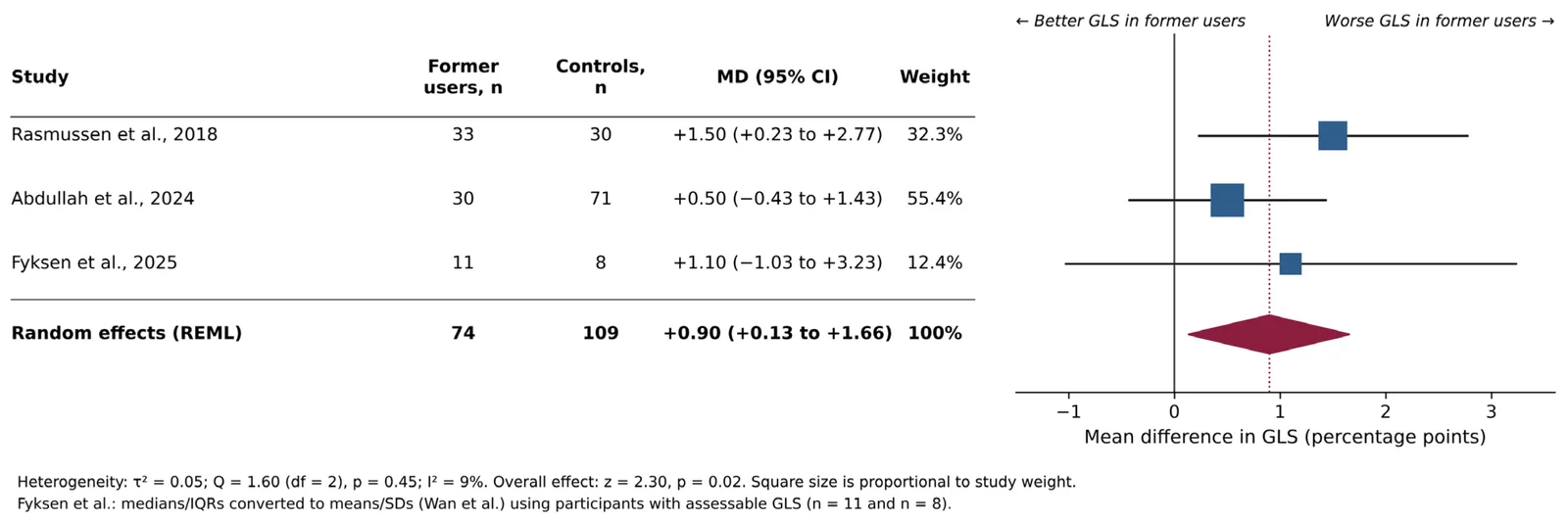
Meta-analysis of global longitudinal strain in former anabolic-androgenic steroid users compared with AAS-naïve controls. Forest plot showing mean differences (MDs) in global longitudinal strain (GLS) between former anabolic-androgenic steroid (AAS) users and AAS-naïve controls. Three studies contributed to the random-effects meta-analysis. The pooled MD was +0.90 percentage points (95% CI +0.13 to +1.66), with low between-study heterogeneity (I^2^ = 9%). Because GLS is conventionally expressed as a negative value, a positive mean difference indicates less negative GLS and therefore worse myocardial deformation in former AAS users. Squares represent study-specific estimates, with their size proportional to study weight; horizontal lines indicate 95% CIs, and the diamond represents the pooled estimate.

Three independent studies contributed to the global longitudinal strain analysis among former/discontinued AAS users [4,5,23]. Former users had a significantly less negative global longitudinal strain than AAS-naïve controls (MD +0.90 percentage points, 95% CI +0.13 to +1.66; *p* = 0.02), with low statistical heterogeneity (I^2^ = 9%; τ^2^ = 0.05; Q = 1.60, *p* = 0.45) (Figure 4). The estimate was sensitive to individual studies: in leave-one-out analyses, it was no longer statistically significant after omitting Rasmussen et al. (MD +0.60, 95% CI −0.26 to +1.45) or Fyksen et al. (MD +0.90, 95% CI −0.06 to +1.86). Because a less negative value reflects worse longitudinal deformation, the pooled estimate indicates a residual between-group difference in subclinical systolic function rather than an individual recovery trajectory. The three studies represented markedly different intervals since cessation: approximately 30 months in Rasmussen et al., approximately 6 years in Abdullah et al., and a median of 12 years in the discontinued subgroup of the Fyksen follow-up [4,5,23]. Cessation ascertainment and imaging protocols also differed across studies, contributing to the very low certainty rating.

Only two studies contributed to the left ventricular ejection fraction meta-analysis, and their individual estimates diverged substantially (Abdullah et al.: MD −9.00 percentage points; Fyksen et al.: MD −1.00 percentage point), yielding a pooled difference that was not statistically significant and was highly heterogeneous (MD −4.97 percentage points, 95% CI −12.81 to +2.87; *p* = 0.21; I^2^ = 96%) (Supplementary Figure S1). The pooled estimate was retained solely as an exploratory quantitative summary because pooling of left ventricular ejection fraction was prespecified in the registered protocol and both studies reported the outcome in common units; however, given the extreme heterogeneity and small evidence base, the individual study estimates are more informative than the pooled value, and no common recovery effect should be inferred. Certainty was very low for both post-cessation outcomes.

### 3.6. Qualitative Synthesis: Mechanisms, Sudden Death, and Effect Modifiers

Across imaging and pathological studies, recurrent structural abnormalities included increased LV mass, hypertrophy, myocardial fibrosis, necrosis, myocyte disarray, and systolic dysfunction [2,3,4,5,7,9,10,23]. Forensic series linked these abnormalities with sudden cardiac death and documented frequent concomitant use of psychoactive substances or multiple performance-enhancing drugs [7,8,9,10]. Competitive bodybuilding cohorts reported substantial sudden cardiac death incidence, particularly among professional male athletes, but individual AAS exposure was not systematically verified, so these cohorts were not pooled with confirmed-exposure epidemiological studies [24,25]. Pharmacovigilance data additionally suggest that stacking AAS with other agents is associated with more severe outcomes, supporting polypharmacy as an important effect modifier [26]. A structured qualitative synthesis is provided in Supplementary Table S4.

### 3.7. Results of Risk-of-Bias and GRADE Assessments

ROBINS-E identified confounding and selection into the exposed cohorts as the dominant limitations of the mortality evidence. Overall risk of bias was judged high for Petersson et al. [12], Thiblin et al. [13], and Windfeld-Mathiasen et al. (2024) [15]. Objective analytical testing strengthened exposure classification in Petersson and Thiblin, whereas the Danish cohort provided robust registry-based outcome ascertainment but retained residual confounding and some uncertainty in exposure classification because anti-doping sanctions could also follow refusal to provide a urine sample. The post-cessation imaging studies (Rasmussen et al. [4], Abdullah et al. [5], and Fyksen et al. 2025 [23]) were also judged overall at high risk of bias, chiefly because of selection into small follow-up samples and residual confounding by training, diet, and concurrent pharmacological exposures. Study-level, domain-specific ROBINS-E judgements are provided in Supplementary Table S5. These limitations were reflected in the GRADE assessment (Table 2).

## 4. Discussion

This systematic review and meta-analysis provide three clinically complementary findings. First, individually ascertained AAS exposure was associated with an approximately 2.6-fold higher risk of all-cause mortality across three observational cohorts, although the evidence remains limited by residual confounding, differing follow-up durations, and the small number of studies. Second, a large contemporary Danish matched cohort reported associations across a broad range of cardiovascular outcomes, with particularly large estimates for cardiomyopathy and heart failure; these findings require independent replication. Third, former AAS users showed a small residual impairment in global longitudinal strain compared with AAS-naïve controls, but the evidence was of very low certainty and does not establish individual recovery trajectories.

### 4.1. All-Cause Mortality: Moving Beyond Surrogate Phenotypes

Much of the earlier AAS cardiovascular literature focused on intermediate phenotypes such as left ventricular hypertrophy, reduced strain, altered lipids, erythrocytosis, and blood pressure [1,2,3,4,5]. The present synthesis extends this evidence to all-cause mortality. The pooled event-based association was robust to leave-one-out, fixed-effect, and exposure-ascertainment sensitivity analyses. However, the wide prediction interval and the small number of cohorts emphasize that precision about the magnitude of the association in a future population remains limited.

These findings should be interpreted alongside the Danish cohort reported by Windfeld-Mathiasen et al., which provided population-based evidence of increased mortality among men receiving AAS-related anti-doping sanctions [15]. The present meta-analysis complements that observation by showing a similar direction of association in two analytically confirmed Swedish cohorts. Restricting the synthesis to those two cohorts yielded RR 2.23 (95% CI 1.50–3.31), while the adjusted HR in Thiblin et al. and the age-matched HR in Windfeld-Mathiasen et al. were also directionally consistent with the pooled event-based RRs. The contribution of the present analysis is therefore to quantify the available comparative evidence and examine its robustness, not to establish causality.

The absence of statistical heterogeneity should not be interpreted as proof of clinical comparability or a direct causal effect. The three cohorts arose from different settings: a clinically referred testing population, a nationwide population of tested individuals, and a Danish anti-doping sanction cohort. Baseline risk, exposure ascertainment, comparator selection, and follow-up duration therefore differed materially. In addition, the pooled RRs are based on cumulative event counts and do not account for person-time, while residual confounding by psychiatric morbidity, polysubstance use, risk-taking behaviour, socioeconomic factors, and other performance-enhancing exposures remains substantial [8,12,15,22]. The broad 95% prediction interval further underscores uncertainty associated with the small number of studies.

### 4.2. A Coherent Cardiovascular Phenotype

The clinical effect-size profile is biologically coherent with the structural literature: cardiomyopathy showed the strongest reported association, followed by heart failure and coronary disease, while VTE and arrhythmia were also increased [6]. Potential mechanisms include androgen-receptor-mediated myocardial hypertrophy, hypertension, adverse lipid changes, erythrocytosis and procoagulant effects, endothelial dysfunction, accelerated atherosclerosis, inflammation, extracellular matrix remodelling, and direct myocardial injury [1,3,9,10]. The recurring forensic findings of fibrosis, necrosis, and myocyte disarray provide a plausible substrate for ventricular dysfunction and electrical instability [7,9,10], and, taken together, these observations suggest that the cardiovascular associations of AAS exposure may extend across myocardial, coronary, thrombotic, and arrhythmic disease domains.

### 4.3. Cardiac Function in Former AAS Users After Cessation

Rasmussen et al. reported worse systolic indices in former users, Abdullah et al. identified severe biventricular dysfunction in both current and former long-term users, and the 16-year Fyksen follow-up documented improvement in some structural and functional measures after discontinuation [4,5,23]. The pooled global longitudinal strain analysis addresses a different question: it quantifies residual group differences between former users and AAS-naïve controls at follow-up. It should not be interpreted as a longitudinal estimate of myocardial recovery because the studies assessed participants at different intervals after cessation and did not provide a common within-person pre-cessation-to-post-cessation trajectory.

The left ventricular ejection fraction synthesis should be interpreted with even greater caution. It rests on only two studies with markedly discordant estimates and extreme heterogeneity (I^2^ = 96%); accordingly, the pooled value is presented as exploratory, and the individual study results are emphasized. Global longitudinal strain may detect subtle myocardial dysfunction when ejection fraction appears preserved, but the small number of observational studies, variable cessation intervals, differences in abstinence ascertainment, and residual confounding mean that persistent impairment cannot be assumed for all former users. Whether the observed residual strain difference predicts future heart failure, arrhythmia, or mortality remains unknown and requires prospective longitudinal study.

### 4.4. Clinical Implications

Non-medical AAS use should be actively considered in the cardiovascular history, especially in younger or middle-aged individuals with otherwise unexplained LV hypertrophy, cardiomyopathy, premature coronary disease, venous thromboembolism, or arrhythmia. A generic question about prescribed medications is unlikely to capture illicit or performance-enhancing exposure; clinicians may need to ask specifically about AAS cycles, cumulative duration, stacking, stimulants, growth hormone, thyroid agents, and recreational drugs.

The EAPC position statement emphasizes the cardiovascular risks of doping substances and the importance of recognizing exposure in athletes and physically active individuals [1], but current evidence is insufficient to mandate a universal screening algorithm. Nevertheless, blood pressure, lipid profile, hematocrit/hemoglobin, electrocardiography, and echocardiography are reasonable components of targeted clinical evaluation, and GLS may be particularly informative when LVEF is preserved despite significant exposure or symptoms. Former users with persistent abnormalities may merit longitudinal follow-up, although optimal surveillance intervals remain undefined.

### 4.5. Strengths and Limitations

The principal strength of this review is its deliberate separation of exposure certainty and cohort overlap. Historical power-sport participation was not treated as equivalent to analytically confirmed AAS exposure, forensic cases were distinguished from denominator-based cohorts, and overlapping Danish publications were assigned to the most informative outcome domains. In addition, post-cessation myocardial function was analysed separately from the broader literature on current AAS use.

The limitations are substantial. All pooled evidence was observational. Residual confounding by recreational drugs, stimulants, alcohol, psychiatric morbidity, socioeconomic factors, extreme resistance training, diet, dehydration, and other performance-enhancing agents cannot be excluded. AAS dose, purity, and cumulative duration were inconsistently reported. The mortality meta-analysis contained only three cohorts from clinically different settings, two of which (both Swedish) may not be fully independent, precluding reliable assessment of publication bias or meta-regression. Although I^2^ and τ^2^ were zero, the heterogeneity statistics have limited power with only three studies and should not be interpreted as evidence that the populations are methodologically or clinically equivalent. The 95% prediction interval was correspondingly wide. The primary mortality synthesis used unadjusted event-based RRs to preserve a common effect measure; these estimates do not account for differing follow-up durations and discard the available confounder adjustment, so the available time-to-event estimates (one confounder-adjusted and one age-matched) were reported alongside them and should be given greater interpretive weight. Post-cessation analyses were small, the pooled global longitudinal strain difference was sensitive to the omission of individual studies, and the analyses compared former users with controls at different intervals after cessation rather than estimating common within-person recovery trajectories. One study required conversion of non-parametric summary data using an established approximation method [21]. The pooled left ventricular ejection fraction estimate rests on only two highly heterogeneous studies and is exploratory. Most participants were men, limiting generalizability to women. The review process also has limitations: searches were restricted to four bibliographic databases without dedicated grey-literature or dissertation searching, and the original line-by-line database search histories and database-specific retrieval totals were not retained. The Supplementary Methods therefore present transparent reconstructed final-update strategies rather than verbatim archival search histories. This limits exact reproducibility of the original retrieval process and should be considered when interpreting the comprehensiveness of the review. Reliance on published aggregate data, rather than individual participant data, also limited the granularity of confounding adjustment.

### 4.6. Research Priorities

Prospective multicentre cohorts should standardize exposure ascertainment, cumulative dose metrics, stacking and concomitant drug documentation, and serial cardiovascular phenotyping before and after cessation. Linkage to national cardiovascular and mortality registries would permit direct testing of whether imaging abnormalities mediate hard outcomes. Female users require dedicated study. A core outcome set for AAS cardiovascular research would improve comparability and reduce selective reporting.

## 5. Conclusions

Confirmed non-medical AAS exposure was associated with higher all-cause mortality across three observational cohorts. The association was robust to several sensitivity analyses, but the small number of clinically different populations, differing follow-up durations, event-based pooling, and residual confounding preclude causal inference. A single large matched Danish cohort also reported higher risks across several cardiovascular outcomes, particularly cardiomyopathy and heart failure, findings that require independent replication. Former AAS users showed worse global longitudinal strain than AAS-naïve controls, although the certainty of the evidence was very low and the available studies do not establish individual recovery trajectories. These findings support recognition of non-medical AAS use as an important marker of cardiovascular risk and reinforce the need for prospective longitudinal studies with standardized exposure ascertainment and follow-up.

## Figures and Tables

**Table 1:** Characteristics and role in evidence synthesis of primary studies included in the systematic review.

Study	Country	Design/Population	Exposure Ascertainment	Comparator	Follow-Up/Observation	Principal Contribution	Role in Synthesis
Pärssinen et al. 2000 [11]	Finland	Retrospective historical cohort; 62 elite male powerlifters	Probable historical exposure; individual AAS use not verified	1094 population controls	12 y	All-cause and cause-specific mortality	Historical/proxy-exposure sensitivity evidence
Petersson et al. 2006 [12]	Sweden	Controlled retrospective cohort; 248 AAS-positive participants	Analytically confirmed positive AAS test	1215 AAS-negative tested participants	From first test (1995–2001) to June 2002	All-cause mortality	Primary mortality meta-analysis †
Darke et al. 2014 [8]	Australia	Forensic case series; 24 AAS-positive sudden or unnatural deaths	AAS metabolites and/or abnormal testosterone/epitestosterone ratio in post-mortem urine	None; no population denominator	1996–2012 case ascertainment	Cause/manner of death, toxicology, polypharmacy and autopsy cardiovascular pathology	Qualitative forensic/mechanistic evidence
Lindqvist et al. 2014 [22]	Sweden	Historical cohort; 1133 former elite male power-sport athletes	Probable historical exposure; individual AAS use not verified	Swedish male population	Long-term	Mortality and suicide	Contextual historical mortality evidence
Thiblin et al. 2015 [13]	Sweden	National cohort; 409 AAS-positive men	≥1 analytically positive AAS test	1604 AAS-negative tested men	Mean 4.3 y	Mortality and cardiovascular morbidity	Primary mortality meta-analysis †
Rasmussen et al. 2018 [4]	Denmark	Cross-sectional cardiac imaging study; current and former AAS users	History of illicit AAS use	Resistance-trained AAS-naïve controls	Former users ≈30 mo after cessation	Cardiac systolic function after cessation; GLS	Post-cessation GLS meta-analysis †
Horwitz et al. 2019 [14]	Denmark	Matched cohort; 545 AAS users	Positive anti-doping test/anti-doping ascertainment	5450 age-matched population controls	Mean 7.4 y	Earlier Danish mortality and morbidity analysis	Qualitative/supportive; excluded from mortality pooling because of cohort overlap
Fyksen et al. 2022 [3]	Norway	Cross-sectional cardiovascular study; 51 long-term AAS users and 21 strength-trained non-users	AAS use/non-use verified by blood and urine analyses; users had ≥3 y cumulative exposure	21 strength-trained competing non-users	Cross-sectional	Cardiovascular phenotype, myocardial remodelling, LVEF and coronary atherosclerosis	Qualitative cardiovascular phenotype; source cohort for later follow-up
Abdullah et al. 2024 [5]	Norway	Cross-sectional imaging study; 101 long-term AAS users	Documented long-term AAS use	71 weightlifting controls	Former users: 6 ± 6 y since cessation	Biventricular function and post-cessation LVEF/GLS	Post-cessation GLS and LVEF meta-analyses †
Windfeld-Mathiasen et al. 2024 [15]	Denmark	Registry matched cohort; 1189 AAS users	National anti-doping ascertainment/sanction	59,450 age-matched population controls	Mean ≈11 y	All-cause, natural and unnatural mortality	Primary mortality meta-analysis †
Windfeld-Mathiasen et al. 2025 [6]	Denmark	Registry matched cohort; 1189 AAS users	National anti-doping ascertainment/sanction	59,450 age-matched population controls	Mean ≈11 y	MI, revascularization, VTE, arrhythmia, cardiomyopathy and heart failure	Supportive major cardiovascular outcomes; same Danish source cohort, not pooled across outcomes
Fyksen et al. 2025 [23]	Norway	Prospective long-term follow-up; 32 AAS users and 13 non-users	AAS use/non-use verified by blood and urine analyses	Strength-trained non-users	Median 16 y in users; 13 y in non-users	Long-term cardiac structure/function and recovery after cessation	Post-cessation GLS and LVEF meta-analyses †; extended follow-up of 2022 source cohort
Vecchiato et al. 2025 [24]	International	Cohort of 20,286 male competitive bodybuilders	AAS exposure not systematically ascertained at individual level	Internal professional/amateur and temporal subgroups	190,211 athlete-years	Mortality and sudden cardiac death incidence	Contextual bodybuilding/SCD evidence
Vecchiato et al. 2026 [25]	International	Cohort of 9447 female competitive bodybuilders	AAS exposure not systematically ascertained at individual level	Internal/sex-specific subgroup comparisons	2005–2020	Mortality and sudden cardiac death incidence in women	Contextual sex-specific bodybuilding/SCD evidence
Heo et al. 2026 [26]	FAERS (USA)	Pharmacovigilance analysis of intentional AAS-misuse reports	Reported intentional AAS misuse in FAERS	No population denominator; stacking vs. non-stacking analyses	Cross-sectional pharmacovigilance	Polypharmacy/stacking and serious or fatal reported outcomes	Qualitative pharmacovigilance evidence

† Contributed to at least one quantitative synthesis. AAS, anabolic-androgenic steroids; FAERS, FDA Adverse Event Reporting System; GLS, global longitudinal strain; LVEF, left ventricular ejection fraction; MI, myocardial infarction; SCD, sudden cardiac death; VTE, venous thromboembolism.

**Table 2:** GRADE certainty-of-evidence assessment and Summary of Findings.

Outcome	Studies	Effect Estimate (95% CI)	Risk of Bias	Inconsistency	Indirectness	Imprecision	Publication Bias	Certainty	Rationale for Certainty Rating
All-cause mortality in confirmed AAS users	3 cohorts	RR 2.57 (1.98–3.33)	Very serious	Not serious (I^2^ = 0%)	Not serious	Not serious	Cannot be formally assessed	⊕⊕◯◯ Low	Observational evidence with residual confounding and exposure-selection concerns. Effect estimates were consistent and reasonably precise; no additional downgrade for inconsistency or imprecision. Rated down two levels for risk of bias.
GLS in former users vs. AAS-naïve controls	3 studies	MD +0.90 pp (+0.13 to +1.66)	Very serious	Not serious (I^2^ = 9%)	Not serious	Serious	Cannot be formally assessed	⊕◯◯◯ Very low	Observational imaging studies with risk of confounding/selection bias and small total sample size. Statistical heterogeneity was minimal, but certainty was limited by imprecision and sparse evidence. Rated down two levels for risk of bias and one level for imprecision.
LVEF in former users vs. AAS-naïve controls	2 studies	MD −4.97 pp (−12.81 to +2.87)	Very serious	Very serious (I^2^ = 96%)	Not serious	Very serious	Cannot be formally assessed	⊕◯◯◯ Very low	Only two small observational studies contributed. Effects were highly heterogeneous and the pooled CI was wide and crossed the null; this synthesis should be regarded as exploratory. Rated down for risk of bias, inconsistency, and imprecision.
Major cardiovascular outcomes (single matched cohort; not pooled)	1 large matched cohort	Adjusted HRs 2.26–8.90 across reported outcomes *	Very serious	Not assessable	Not serious	Serious for less frequent outcomes	Not assessable	⊕◯◯◯ Very low	Evidence derives from one observational matched cohort. Associations were adjusted but remain susceptible to residual confounding; precision varied across distinct cardiovascular endpoints and no pooled estimate was calculated. Rated down two levels for risk of bias and one level for imprecision; inconsistency could not be assessed with a single cohort.

* The range 2.26–8.90 represents adjusted HR point estimates across distinct cardiovascular outcomes and is not a pooled effect estimate. Certainty was rated with GRADE for ROBINS-E–assessed exposure studies, in which evidence starts at high certainty; risk of bias was rated very serious because all contributing studies were judged at high overall risk of bias (Supplementary Table S5; for major cardiovascular outcomes, the judgement for the same Danish source cohort was applied). GRADE certainty: ⊕⊕◯◯, low; ⊕◯◯◯, very low. AAS, anabolic-androgenic steroids; CI, confidence interval; GLS, global longitudinal strain; HR, hazard ratio; LVEF, left ventricular ejection fraction; MD, mean difference; pp, percentage points; RR, risk ratio.

## Data Availability

All study-level data used in the quantitative syntheses were extracted from published reports. All study-level input data, transformations, and derived estimates used in the quantitative syntheses are reported in Supplementary Tables S1–S3. The standardized data-extraction form and the analytic code (SPSS syntax and Python scripts) used to generate the pooled estimates and figures are available from the corresponding author upon reasonable request. Additional materials are available from the corresponding author upon reasonable request.

## Supplementary Materials

The following supporting information can be downloaded at: https://www.mdpi.com/article/10.3390/medsci14060678/s1, Table S1: Cohort overlap, duplicate publications, and rules for quantitative synthesis; Table S2: Study-level quantitative data, transformations, absolute mortality rates, and sensitivity analyses; Table S3: Major cardiovascular outcomes associated with AAS exposure in the expanded Danish Anti-Doping cohort; Table S4: Integrated qualitative synthesis of cardiovascular injury, mortality patterns, post-cessation recovery, and potential effect modifiers; Table S5: Study-level ROBINS-E risk-of-bias assessment; Figure S1: Left ventricular ejection fraction in former anabolic-androgenic steroid users compared with AAS-naïve controls; File S1: PRISMA 2020 checklist; File S2: Supplementary Methods—database-specific search strategies. References [2,3,4,5,6,7,8,9,10,12,13,14,15,16,21,23,24,25,26] are cited in the Supplementary Materials.
