# Supplementary material for: Anabolic–Androgenic Steroid Use, All-Cause Mortality, and Major Cardiovascular Outcomes: A Systematic Review and Meta-Analysis
Source: Med Sci (Basel). 2026 Oct 15;14(6):678. doi: 10.3390/medsci14060678 (PMC13707093; doi:10.3390/medsci14060678)
Supplement: Supplementary file 1 [file medsci-14-00678-s001.zip › File S1 PRISMA_2020_Checklist.pdf]

PRISMA 2020 Checklist

Anabolic-Androgenic Steroid Use, All-Cause Mortality, and Major Cardiovascular Outcomes: A Systematic Review and Meta-Analysis

Del Río-Solá ML, Martin-Hernandez J, Santos-Lozano A. — Med. Sci. (Medical Sciences, MDPI)

From: Page MJ, McKenzie JE, Bossuyt PM, Boutron I, Hoffmann TC, Mulrow CD, et al. The PRISMA 2020 statement: an updated guideline for reporting systematic reviews. BMJ 2021;372:n71.

Section/Topic	Item #	Checklist item (PRISMA 2020)	Reported	Location in manuscript
**TITLE**				
Title	1	Identify the report as a systematic review.	**Yes**	Title.
**ABSTRACT**				
Abstract	2	See the PRISMA 2020 for Abstracts checklist.	**Yes**	Structured abstract (Background/Objectives, Methods, Results, Conclusions).
**INTRODUCTION**				
Rationale	3	Describe the rationale for the review in the context of existing knowledge.	**Yes**	Section 1, Introduction.
Objectives	4	Provide an explicit statement of the objective(s) or question(s) the review addresses.	**Yes**	Section 1, final paragraph (aims i–iv).
**METHODS**				
Eligibility criteria	5	Specify the inclusion and exclusion criteria for the review and how studies were grouped for the syntheses.	**Yes**	Section 2.2 (Eligibility criteria), including stricter criteria for each quantitative synthesis.
Information sources	6	Specify all databases, registers, websites, organisations, reference lists and other sources searched or consulted to identify studies. Specify the date when each source was last searched or consulted.	**Yes**	Section 2.3 (MEDLINE/PubMed, Embase, Scopus, Web of Science; reference lists; last searched 30 August 2026).
Search strategy	7	Present the full search strategies for all databases, registers and websites, including any filters and limits used.	**Yes**	Section 2.3 and Supplementary Methods (full reconstructed final-update strategy for each database; see limitation in Section 4.5).
Selection process	8	Specify the methods used to decide whether a study met the inclusion criteria of the review, including how many reviewers screened each record and each report retrieved, whether they worked independently, and if applicable, details of automation tools used in the process.	**Yes**	Section 2.4 (two independent reviewers; third-reviewer adjudication).
Data collection process	9	Specify the methods used to collect data from reports, including how many reviewers collected data from each report, whether they worked independently, any processes for obtaining or confirming data from study investigators, and if applicable, details of automation tools used in the process.	**Yes**	Section 2.4 (independent duplicate extraction with a standardized form).
Data items – outcomes	10a	List and define all outcomes for which data were sought. Specify whether all results compatible with each outcome domain were sought, and if not, the methods used to decide which results to collect.	**Yes**	Section 2.5 (Outcomes).
Data items – other variables	10b	List and define all other variables for which data were sought (e.g., participant and intervention characteristics, funding sources). Describe any assumptions made about missing or unclear information.	**Yes**	Section 2.4 (extracted variables).

Section/Topic	Item #	Checklist item (PRISMA 2020)	Reported	Location in manuscript
Study risk of bias assessment	11	Specify the methods used to assess risk of bias in the included studies, including details of the tool(s) used, how many reviewers assessed each study and whether they worked independently, and if applicable, details of automation tools used.	Yes	Section 2.6 (ROBINS-E; two independent reviewers; third-reviewer adjudication).
Effect measures	12	Specify for each outcome the effect measure(s) (e.g., risk ratio, mean difference) used in the synthesis or presentation of results.	Yes	Section 2.7 (risk ratios for mortality; mean differences for GLS/LVEF; adjusted hazard ratios for major cardiovascular outcomes).
Synthesis methods – eligibility for each synthesis	13a	Describe the processes used to decide which studies were eligible for each synthesis.	Yes	Sections 2.2 and 2.4 (eligibility for each synthesis; overlap rules; Supplementary Table S1).
Synthesis methods – data preparation	13b	Describe any methods required to prepare the data for presentation or synthesis, such as handling of missing summary statistics or data conversions.	Yes	Section 2.7 (SE-to-SD conversion; median/IQR conversion by the method of Wan et al.); Supplementary Table S2.
Synthesis methods – tabulation/visual display	13c	Describe any methods used to tabulate or visually display results of individual studies and syntheses.	Yes	Table 1, Table 2, Figures 2–4, Supplementary Figure S1, Supplementary Tables S2–S3.
Synthesis methods – method for synthesis	13d	Describe any methods used to synthesize results and provide a rationale. If meta-analysis was performed, describe the model(s), method(s) to identify heterogeneity, and software used.	Yes	Section 2.7 (inverse-variance random-effects model with REML; software).
Synthesis methods – exploring heterogeneity	13e	Describe any methods used to explore possible causes of heterogeneity among study results (e.g., subgroup analysis, meta-regression).	Yes	Section 2.7 (Q, I^2, τ^2; narrative exploration of heterogeneity sources).
Synthesis methods – sensitivity analyses	13f	Describe any sensitivity analyses conducted to assess robustness of the synthesized results.	Yes	Section 2.7 (prespecified leave-one-out; post hoc fixed-effect, prediction interval, ascertainment and independence analyses).
Reporting bias assessment	14	Describe any methods used to assess risk of bias due to missing results in a synthesis (reporting biases).	Yes	Section 2.7 (funnel plots and small-study tests not performed because <10 studies per synthesis).
Certainty assessment	15	Describe any methods used to assess certainty (confidence) in the body of evidence for an outcome.	Yes	Section 2.6 (GRADE); Table 2 footnote.
**RESULTS**				
Study selection	16a	Describe the results of the search and selection process, from the number of records identified to the number of studies included, ideally using a flow diagram.	Yes	Section 3.1 and Figure 1.
Study selection – excluded studies	16b	Cite studies that might appear to meet the inclusion criteria but were excluded, and explain why.	Yes	Figure 1 (full-text exclusion reasons); Section 3.2 and Supplementary Table S1 (overlapping reports).
Study characteristics	17	Cite each included study and present its characteristics.	Yes	Table 1; Section 3.2.
Risk of bias in studies	18	Present assessments of risk of bias for each included study.	Yes	Section 3.7 and Supplementary Table S5.

Section/Topic	Item #	Checklist item (PRISMA 2020)	Reported	Location in manuscript
Results of individual studies	19	For all outcomes, present for each study: summary statistics for each group and an effect estimate with precision, ideally using structured tables or plots.	Yes	Figures 2–4, Supplementary Figure S1, Supplementary Tables S2–S3.
Results of syntheses – characteristics/RoB summary	20a	Briefly summarise the characteristics and risk of bias among studies contributing to the synthesis.	Yes	Sections 3.2–3.5 and 3.7; Table 2.
Results of syntheses – present results	20b	Present results of all statistical syntheses conducted, with summary estimate, precision and heterogeneity measures; describe direction of effect.	Yes	Sections 3.3–3.5; Figures 2–4; Supplementary Figure S1.
Results of syntheses – heterogeneity	20c	Present results of all investigations of possible causes of heterogeneity among study results.	Yes	Sections 3.3 and 3.5 (Q, I^2, τ^2, prediction interval; narrative exploration).
Results of syntheses – sensitivity analyses	20d	Present results of all sensitivity analyses conducted.	Yes	Section 3.3 (mortality) and Section 3.5 (GLS); Supplementary Table S2.
Reporting biases	21	Present assessments of risk of bias due to missing results (reporting biases) for each synthesis assessed.	Yes	Table 2 (publication bias not formally assessable); Section 4.5.
Certainty of evidence	22	Present assessments of certainty (confidence) in the body of evidence for each outcome assessed.	Yes	Table 2 and Section 3.7.
**DISCUSSION**				
Discussion – interpretation	23a	Provide a general interpretation of the results in the context of other evidence.	Yes	Sections 4.1–4.4.
Discussion – limitations of evidence	23b	Discuss any limitations of the evidence included in the review.	Yes	Section 4.5.
Discussion – limitations of review process	23c	Discuss any limitations of the review processes used.	Yes	Section 4.5 (database coverage; reconstructed search strategies; aggregate data).
Discussion – implications	23d	Discuss implications of the results for practice, policy, and future research.	Yes	Sections 4.4, 4.6, and 5.
**OTHER INFORMATION**				
Registration	24a	Provide registration information for the review, including register name and registration number, or state that the review was not registered.	Yes	Section 2.1 (PROSPERO CRD420261518294).
Protocol	24b	Indicate where the review protocol can be accessed, or state that a protocol was not prepared.	Yes	Section 2.1 (PROSPERO record URL).
Amendments	24c	Describe and explain any amendments to information provided at registration or in the protocol.	Yes	Section 2.1 (no protocol amendments; post hoc sensitivity analyses identified as such).
Support	25	Describe sources of financial or non-financial support for the review, and the role of the funders/sponsors.	Yes	Funding statement.
Competing interests	26	Declare any competing interests of review authors.	Yes	Conflicts of Interest statement.
Availability of data, code and materials	27	Report which of the following are publicly available and where: data collection forms, extracted data, analysis data, analytic code, other materials.	Yes	Data Availability Statement; Supplementary Tables S1–S3.
