# Supplementary material for: Anabolic–Androgenic Steroid Use, All-Cause Mortality, and Major Cardiovascular Outcomes: A Systematic Review and Meta-Analysis
Source: Med Sci (Basel). 2026 Oct 15;14(6):678. doi: 10.3390/medsci14060678 (PMC13707093; doi:10.3390/medsci14060678)
Supplement: Supplementary file 1 [file medsci-14-00678-s001.zip › File S2 Supplementary Methods Search Strategies.pdf]

Supplementary Methods. Electronic search strategies

Methodological transparency statement: The original line-by-line database search histories and reliable database-specific retrieval totals were not retained. The strategies below are reconstructed final-update strategies prepared to reflect the concepts and syntax used in the review and to make the final update as reproducible as possible. They should not be interpreted as verbatim archival copies of the initial searches. This limitation is acknowledged explicitly in the main manuscript (Section 4.5).

Review: Anabolic-Androgenic Steroid Use, All-Cause Mortality, and Major Cardiovascular Outcomes: A Systematic Review and Meta-Analysis

Final search-update date: 30 August 2026

Search concepts. The strategy combines terms for non-medical anabolic-androgenic steroid exposure with mortality, sudden death, major cardiovascular outcomes, myocardial disease, vascular/thrombotic outcomes, cardiac imaging/function, and cessation or former use. No language restriction was applied at the search stage; eligibility of human studies was determined during screening.

MEDLINE/PubMed
Search string

(("Anabolic Agents"[Mesh] OR "Androgens"[Mesh] OR "anabolic androgenic steroid"[Title/Abstract] OR "anabolic-androgenic steroid"[Title/Abstract] OR "anabolic androgenic steroids"[Title/Abstract] OR "anabolic-androgenic steroids"[Title/Abstract] OR "anabolic steroid"[Title/Abstract] OR "anabolic steroids"[Title/Abstract] OR AAS[Title/Abstract]) AND (misuse[Title/Abstract] OR abuse[Title/Abstract] OR illicit[Title/Abstract] OR nonmedical[Title/Abstract] OR "non-medical"[Title/Abstract] OR doping[Title/Abstract] OR bodybuilding[Title/Abstract] OR bodybuilder*[Title/Abstract] OR powerlift*[Title/Abstract] OR "strength train*"[Title/Abstract] OR athlete*[Title/Abstract] OR user*[Title/Abstract])) AND ("Mortality"[Mesh] OR "Death, Sudden, Cardiac"[Mesh] OR "Cardiovascular Diseases"[Mesh] OR "Heart Failure"[Mesh] OR "Cardiomyopathies"[Mesh] OR "Myocardial Infarction"[Mesh] OR "Arrhythmias, Cardiac"[Mesh] OR "Venous Thromboembolism"[Mesh] OR mortality[Title/Abstract] OR death[Title/Abstract] OR "sudden death"[Title/Abstract] OR "sudden cardiac death"[Title/Abstract] OR cardiovascular[Title/Abstract] OR "myocardial infarction"[Title/Abstract] OR coronary[Title/Abstract] OR atherosclero*[Title/Abstract] OR thrombosis[Title/Abstract] OR thromboembol*[Title/Abstract] OR arrhythmia*[Title/Abstract] OR cardiomyopath*[Title/Abstract] OR "heart failure"[Title/Abstract] OR echocardiograph*[Title/Abstract] OR "ejection fraction"[Title/Abstract] OR LVEF[Title/Abstract] OR "global longitudinal strain"[Title/Abstract] OR GLS[Title/Abstract] OR "myocardial flow reserve"[Title/Abstract] OR fibrosis[Title/Abstract] OR cessation[Title/Abstract] OR discontinu*[Title/Abstract] OR "former user*"[Title/Abstract] OR "past user*"[Title/Abstract]))

Sentinel included reports retrieved by the reconstructed PubMed strategy were Petersson et al. (2006), Rasmussen et al. (2018), Windfeld-Mathiasen et al. (2024, 2025), Abdullah et al. (2024), and Fyksen et al. (2025).

Embase (Elsevier syntax)

('anabolic agent'/exp OR 'androgen'/exp OR 'anabolic androgenic steroid':ti,ab,kw OR 'anabolic-androgenic steroid':ti,ab,kw OR 'anabolic androgenic steroids':ti,ab,kw OR 'anabolic-androgenic steroids':ti,ab,kw OR 'anabolic steroid':ti,ab,kw OR 'anabolic steroids':ti,ab,kw OR aas:ti,ab,kw) AND (misuse:ti,ab,kw OR abuse:ti,ab,kw OR illicit:ti,ab,kw OR nonmedical:ti,ab,kw OR 'non-medical':ti,ab,kw OR doping:ti,ab,kw OR bodybuilding:ti,ab,kw OR bodybuilder*:ti,ab,kw OR powerlift*:ti,ab,kw OR 'strength train*':ti,ab,kw OR athlete*:ti,ab,kw OR user*:ti,ab,kw) AND ('mortality'/exp OR 'sudden cardiac death'/exp OR 'cardiovascular disease'/exp OR 'heart failure'/exp OR 'cardiomyopathy'/exp OR 'myocardial infarction'/exp OR 'heart arrhythmia'/exp OR 'venous thromboembolism'/exp OR mortality:ti,ab,kw OR death:ti,ab,kw OR 'sudden death':ti,ab,kw OR 'sudden cardiac death':ti,ab,kw OR cardiovascular:ti,ab,kw OR 'myocardial infarction':ti,ab,kw OR coronary:ti,ab,kw OR atherosclero*:ti,ab,kw OR thrombosis:ti,ab,kw OR thromboembol*:ti,ab,kw OR arrhythmia*:ti,ab,kw OR cardiomyopath*:ti,ab,kw OR 'heart failure':ti,ab,kw OR echocardiograph*:ti,ab,kw OR 'ejection fraction':ti,ab,kw OR lvef:ti,ab,kw OR 'global longitudinal strain':ti,ab,kw OR gls:ti,ab,kw OR 'myocardial flow reserve':ti,ab,kw OR fibrosis:ti,ab,kw OR cessation:ti,ab,kw OR discontinu*:ti,ab,kw OR 'former user*':ti,ab,kw OR 'past user*':ti,ab,kw)

Scopus

TITLE-ABS-KEY(("anabolic androgenic steroid" OR "anabolic-androgenic steroid" OR "anabolic androgenic steroids" OR "anabolic-androgenic steroids" OR "anabolic steroid" OR "anabolic steroids" OR AAS) AND (misuse OR abuse OR illicit OR nonmedical OR "non-medical" OR doping OR bodybuilding OR bodybuilder* OR powerlift* OR "strength train*" OR athlete* OR user*) AND (mortality OR death OR "sudden death" OR "sudden cardiac death" OR cardiovascular OR "myocardial infarction" OR coronary OR atherosclero* OR thrombosis OR thromboembol* OR arrhythmia* OR cardiomyopath* OR "heart failure" OR echocardiograph* OR "ejection fraction" OR LVEF OR "global longitudinal strain" OR GLS OR "myocardial flow reserve" OR fibrosis OR cessation OR discontinu* OR "former user*" OR "past user*"))

Web of Science Core Collection

TS=(("anabolic androgenic steroid" OR "anabolic-androgenic steroid" OR "anabolic androgenic steroids" OR "anabolic-androgenic steroids" OR "anabolic steroid" OR "anabolic steroids" OR AAS) AND (misuse OR abuse OR illicit OR nonmedical OR "non-medical" OR doping OR bodybuilding OR bodybuilder* OR powerlift* OR "strength train*" OR athlete* OR user*) AND (mortality OR death OR "sudden death" OR "sudden cardiac death" OR cardiovascular OR "myocardial infarction" OR coronary OR atherosclero* OR thrombosis OR thromboembol* OR arrhythmia* OR cardiomyopath* OR "heart failure" OR echocardiograph* OR "ejection fraction" OR LVEF OR

"global longitudinal strain" OR GLS OR "myocardial flow reserve" OR fibrosis OR cessation OR discontinu* OR "former user*" OR "past user*"))

Supplementary search procedures

Backward citation searching was performed from all eligible primary reports and from relevant systematic reviews. Forward citation searching was used for key mortality and post-cessation studies. Recent-publication checks focused on 2024-2026 records and on reports describing former AAS users, mortality, cardiovascular events, myocardial function, coronary microcirculation, and forensic cardiac pathology.

Deduplication and screening. Records retrieved from the four databases were combined and deduplicated before screening. The aggregate numbers of records identified (n = 1248) and duplicates removed (n = 386) are reported in Figure 1; these totals cannot be decomposed reliably by database because the original database-level exports were not retained.
