# Supplementary material for: Anabolic–Androgenic Steroid Use, All-Cause Mortality, and Major Cardiovascular Outcomes: A Systematic Review and Meta-Analysis
Source: Med Sci (Basel). 2026 Oct 15;14(6):678. doi: 10.3390/medsci14060678 (PMC13707093; doi:10.3390/medsci14060678)
Supplement: Supplementary file 1 [file medsci-14-00678-s001.zip › Supplementary Table S1.pdf]

Supplementary Table S1. Cohort overlap, duplicate publications, and rules for quantitative synthesis

Cohort cluster	Publication	Population	Overlap	Outcomes	Use in synthesis	Rationale
Danish Anti-Doping cohort	Horwitz 2019 [14]	545 AAS-positive vs 5450 controls	Earlier subset of the Danish source cohort	Mortality; ischaemic/non-ischaemic heart disease; thromboembolism	Qualitative/supportive; not used in primary pooled mortality	Superseded for mortality by the larger and more recent Danish cohort; exclusion prevents double counting.
Danish Anti-Doping cohort	Windfeld-Mathiasen 2024 [15]	1189 AAS users vs 59,450 controls	Expanded Danish cohort	All-cause, natural, and unnatural mortality	Selected for primary mortality meta-analysis	Largest and most recent mortality report from this source cohort.
Danish Anti-Doping cohort	Windfeld-Mathiasen 2025 [6]	Same 1189 AAS users vs 59,450 controls	Same source cohort as Windfeld-Mathiasen 2024	MI; PCI/CABG; VTE; stroke; arrhythmia; cardiomyopathy; HF; cardiac arrest	Narrative quantitative presentation of distinct cardiovascular outcomes; not meta-analysed	Retained for non-overlapping cardiovascular endpoints; mortality data were not counted again.
Swedish AAS-testing cohorts	Petersson 2006 [12]	248 AAS-positive vs 1215 AAS-negative	No participant overlap reported; both cohorts were assembled from individuals tested for AAS in Sweden and shared investigators, so independence could not be verified	Mortality; morbidity	Included in primary mortality meta-analysis	Treated as independent in the primary analysis; potential partial overlap with Thiblin 2015 addressed by a post hoc sensitivity analysis excluding this cohort (RR 2.53, 95% CI 1.84–3.46).
Swedish AAS-testing cohorts	Thiblin 2015 [13]	409 AAS-positive vs 1604 AAS-negative	No participant overlap reported; independence from Petersson 2006 could not be verified (see above)	Mortality; cardiovascular morbidity	Included in primary mortality meta-analysis	Treated as an independent comparative cohort based on the available cohort descriptions and recruitment information.

Cohort cluster	Publication	Population	Overlap	Outcomes	Use in synthesis	Rationale
Norwegian AAS cardiovascular cohort	Fyksen 2022 [3]	51 long-term AAS users vs 21 strength-trained non-users	Earlier assessment of the source cohort	Cardiac structure/function; coronary atherosclerosis	Qualitative cardiovascular phenotype; not separately pooled	Earlier characterization of the cohort subsequently reassessed at long-term follow-up.
Norwegian AAS cardiovascular cohort	Fyksen 2025 [23]	32 AAS users vs 13 non-users at long-term follow-up	Long-term follow-up of the earlier source cohort	Cardiac structure/function and recovery after cessation	Selected for post-cessation GLS and LVEF syntheses	Long-term follow-up provides the relevant cessation/recovery estimates and avoids duplicate use of overlapping participants.
IFBB male bodybuilding cohort	Vecchiato 2025 [24]	20,286 male competitive bodybuilders	Independent of the confirmed-AAS comparative cohorts	Mortality and SCD incidence	Qualitative/contextual synthesis	AAS exposure was not individually ascertained; therefore excluded from causal AAS-effect pooling.
IFBB female bodybuilding cohort	Vecchiato 2026 [25]	9447 female competitive bodybuilders	Independent female cohort using a related bodybuilding framework	Mortality and SCD incidence	Qualitative/contextual synthesis	AAS exposure was not individually ascertained; therefore excluded from causal AAS-effect pooling.

AAS, anabolic-androgenic steroids; CABG, coronary artery bypass grafting; CV, cardiovascular; GLS, global longitudinal strain; HF, heart failure; LVEF, left ventricular ejection fraction; MI, myocardial infarction; PCI, percutaneous coronary intervention; SCD, sudden cardiac death; VTE, venous thromboembolism. Overlapping reports were retained when they contributed distinct outcomes, but participants from the same source cohort were not counted more than once within any quantitative synthesis.

No sequential reports from the same source population contributed simultaneously to the primary all-cause mortality meta-analysis. Potential partial overlap between the two Swedish cohorts could not be excluded and was addressed by a sensitivity analysis excluding Petersson 2006. The 2019, 2024, and 2025 Danish publications were treated as sequential reports from the same source population and assigned to distinct outcome roles to avoid double counting.
