# Supplementary material for: Anabolic–Androgenic Steroid Use, All-Cause Mortality, and Major Cardiovascular Outcomes: A Systematic Review and Meta-Analysis
Source: Med Sci (Basel). 2026 Oct 15;14(6):678. doi: 10.3390/medsci14060678 (PMC13707093; doi:10.3390/medsci14060678)
Supplement: Supplementary file 1 [file medsci-14-00678-s001.zip › Supplementary Table S2.pdf]

Supplementary Table S2. Study-level quantitative data, transformations, absolute mortality rates, and sensitivity analyses

Study	Outcome	AAS/former group	Comparator	Source data	Effect used in meta-analysis	Transformation / derivation
Petersson 2006 [12]	All-cause mortality	12/248	22/1215	Deaths / total participants in each tested group	RR 2.67 (95% CI 1.34–5.33); mortality meta-analysis (Figure 2)	Unadjusted event-based RR calculated from reported 2×2 event data. Exposure analytically confirmed; no directly comparable adjusted HR was available.
Thiblin 2015 [13]	All-cause mortality	24/409	46/1604	Deaths / total participants; adjusted HR 1.69 (95% CI 1.02–2.82) also reported	RR 2.05 (95% CI 1.26–3.31); mortality meta-analysis (Figure 2)	Unadjusted event-based RR calculated from reported 2×2 event data to preserve a common effect measure. Adjusted HR retained as supportive evidence.
Windfeld-Mathiasen 2024 [15]	All-cause mortality	33/1189	578/59,450	Deaths / total participants; age-matched, otherwise unadjusted HR 2.81 (95% CI 1.98–3.99) also reported	RR 2.85 (95% CI 2.02–4.04); mortality meta-analysis (Figure 2)	Unadjusted event-based RR calculated from reported 2×2 event data. Expanded Danish mortality cohort selected to avoid overlap with the earlier report.
Rasmussen 2018 [4]	GLS after cessation	Former users n=33	Controls n=30	Mean GLS −16.7, SE 0.5 vs −18.2, SE 0.4	MD +1.50 pp (95% CI +0.23 to +2.77); GLS meta-analysis (Figure 4)	Reported SEs converted to SDs using SD = SE × √n; group means/SDs used to derive the mean difference and variance.
Abdullah 2024 [5]	GLS after cessation	Former users n=30	Controls n=71	Reported group summary data for former users and controls	MD +0.50 pp, SE 0.47 (95% CI −0.43 to +1.43); GLS meta-analysis (Figure 4)	Mean difference and SE derived from the reported group-level summary statistics.
Abdullah 2024 [5]	LVEF after cessation	Former users n=30	Controls n=71	Reported group summary data for former users and controls	MD −9.00 pp, SE 1.26 (95% CI −11.48 to −6.52); LVEF meta-analysis (Supplementary Figure S1)	Mean difference and SE derived from the reported group-level summary statistics.

Study	Outcome	AAS/former group	Comparator	Source data	Effect used in meta-analysis	Transformation / derivation
Fyksen 2025 [23]	GLS after cessation	Discontinued n=11 (assessable GLS)	Controls n=8 (assessable GLS)	Median (IQR) GLS −16.6% (−19.3 to −15.6) vs −18.3% (−19.1 to −17.4)	MD +1.10 pp, SE 1.09 (95% CI −1.03 to +3.23); GLS meta-analysis (Figure 4)	Median/IQR values were converted to approximate means and SDs using the method of Wan et al. (2014) [21] with the number of participants with assessable GLS; group summaries were then used to derive the mean difference and variance.
Fyksen 2025 [23]	LVEF after cessation	Discontinued n=15	Controls n=13	Median (IQR) LVEF 52% (51 to 55) vs 54% (52 to 55)	MD −1.00 pp, SE 1.09 (95% CI −3.14 to +1.14); LVEF meta-analysis (Supplementary Figure S1)	Median/IQR values were converted to approximate means and SDs using the method of Wan et al. (2014) [21]; group summaries were then used to derive the mean difference and variance.

Unadjusted event-based RRs were used for the primary mortality meta-analysis to preserve a common effect measure across studies. Because these RRs do not account for differences in follow-up duration, time-to-event estimates (confounder-adjusted in Thiblin 2015; age-matched only in Windfeld-Mathiasen 2024) and absolute incidence rates were retained where available and emphasized in interpretation.

A positive GLS mean difference indicates a less negative (worse) strain value in former users relative to controls.

AAS, anabolic-androgenic steroids; CI, confidence interval; GLS, global longitudinal strain; HR, hazard ratio; IQR, interquartile range; LVEF, left ventricular ejection fraction; MD, mean difference; pp, percentage points; RR, risk ratio; SD, standard deviation; SE, standard error.

Sensitivity analyses and absolute mortality rates

Analysis	Estimate	95% CI / prediction interval	Interpretation
Primary random-effects RR	2.57	95% CI 1.98–3.33	Three cohorts; Q=1.23, I^2=0%, τ^2=0.
95% prediction interval	-	0.47–13.87	Higgins-Thompson-Spiegelhalter approach (t distribution, k-2 df); wide because k=3; post hoc.

Fixed-effect sensitivity RR	2.57	95% CI 1.98–3.33	Identical because estimated τ^2=0; post hoc.
Analytically confirmed exposure only	2.23	95% CI 1.50–3.31	Petersson 2006 + Thiblin 2015; excludes Danish anti-doping sanction cohort (equivalent to the prespecified leave-one-out omitting Windfeld-Mathiasen 2024).
Excluding Petersson 2006 (uncertain independence)	2.53	95% CI 1.84–3.46	Thiblin 2015 + Windfeld-Mathiasen 2024; post hoc, because independence of the two Swedish cohorts could not be verified.
Leave-one-out: excluding Thiblin 2015	2.82	95% CI 2.07–3.84	Petersson 2006 + Windfeld-Mathiasen 2024; prespecified.
Thiblin 2015 absolute mortality rate	14.0 vs 6.7 per 1000 person-years	-	AAS-positive vs AAS-negative tested participants.
Windfeld-Mathiasen 2024 absolute mortality rate	2.48 vs 0.88 per 1000 person-years	-	AAS-sanctioned vs matched controls; calculated from reported person-time.
Petersson 2006 absolute mortality rate	Not estimable	-	Person-time not reported in a form permitting comparable rate calculation.
GLS leave-one-out: omitting Rasmussen 2018	MD +0.60 pp	95% CI −0.26 to +1.45	Post-cessation GLS; no longer statistically significant.
GLS leave-one-out: omitting Abdullah 2024	MD +1.40 pp	95% CI +0.30 to +2.49	Post-cessation GLS.
GLS leave-one-out: omitting Fyksen 2025	MD +0.90 pp	95% CI −0.06 to +1.86	Post-cessation GLS; no longer statistically significant.

Reference for conversion method: Wan X, Wang W, Liu J, Tong T. BMC Med Res Methodol. 2014;14:135. doi:10.1186/1471-2288-14-135.
