# Supplementary material for: Anabolic–Androgenic Steroid Use, All-Cause Mortality, and Major Cardiovascular Outcomes: A Systematic Review and Meta-Analysis
Source: Med Sci (Basel). 2026 Oct 15;14(6):678. doi: 10.3390/medsci14060678 (PMC13707093; doi:10.3390/medsci14060678)
Supplement: Supplementary file 1 [file medsci-14-00678-s001.zip › Supplementary Table S3.pdf]

Supplementary Table S3. Major cardiovascular outcomes associated with AAS exposure in the expanded Danish Anti-Doping cohort [6]

Outcome	AAS users, events/N	Controls, events/N	Adjusted hazard ratio (95% CI)
Acute myocardial infarction	12/1189	189/59,450	3.00 (1.67–5.39)
PCI or CABG	13/1189	219/59,450	2.95 (1.68–5.18)
Venous thromboembolism	22/1189	392/59,450	2.42 (1.54–3.80)
Arrhythmia	29/1189	625/59,450	2.26 (1.53–3.32)
Cardiomyopathy	14/1189	77/59,450	8.90 (4.99–15.88)
Heart failure	12/1189	156/59,450	3.63 (2.01–6.55)
Ischaemic stroke	<5/1189	164/59,450	Not reportable
Cardiac arrest	<5/1189	54/59,450	Not reportable

Adjusted hazard ratios are reproduced as reported in the original study [6] and are adjusted for age, diabetes, occupational status, and educational length at baseline; AAS users were men sanctioned for AAS use in the Danish fitness-centre anti-doping programme (2006–2018), matched 1:50 by age to population controls.

No pooled estimate was calculated because these cardiovascular endpoints are clinically distinct and originate from the same source cohort.

For ischaemic stroke and cardiac arrest, fewer than five events occurred among AAS users; the corresponding adjusted hazard ratios were not reportable.

AAS, anabolic-androgenic steroids; CABG, coronary artery bypass grafting; CI, confidence interval; PCI, percutaneous coronary intervention.
