# Supplementary material for: Anabolic–Androgenic Steroid Use, All-Cause Mortality, and Major Cardiovascular Outcomes: A Systematic Review and Meta-Analysis
Source: Med Sci (Basel). 2026 Oct 15;14(6):678. doi: 10.3390/medsci14060678 (PMC13707093; doi:10.3390/medsci14060678)
Supplement: Supplementary file 1 [file medsci-14-00678-s001.zip › Supplementary Table S4.pdf]

Supplementary Table S4. Integrated qualitative synthesis of cardiovascular injury, mortality patterns, post-cessation recovery, and potential effect modifiers

Evidence domain	Primary studies	Supporting secondary evidence	Key findings	Interpretation
Atherosclerotic / ischaemic injury	Fyksen 2022 [3]; Windfeld-Mathiasen 2025 [6]	Di Fazio 2025 [9]	Adverse cardiovascular phenotype and coronary atherosclerosis were reported in long-term users; the Danish cohort showed increased risks of acute MI and coronary revascularization.	Consistent with an atherosclerotic/atherothrombotic pathway, while causal attribution remains limited by observational design and potential co-exposures.
Venous thrombotic injury	Horwitz 2019 [14]; Windfeld-Mathiasen 2025 [6]	—	Venous thromboembolism was over-represented among AAS-exposed participants in Danish cohort analyses.	Consistent with a potential prothrombotic pathway; the biological mechanism was not directly tested in these cohorts.
Pathological myocardial remodelling	Abdullah 2024 [5]; Fyksen 2022 [3]; Fyksen 2025 [23]	Cavalcante 2026 [2]	Long-term AAS exposure was associated with adverse cardiac remodelling and impaired systolic function; follow-up data indicate persistent abnormalities in some former users.	Provides a structural and functional substrate compatible with cardiomyopathy and heart failure.
Direct myocardial injury / fibrosis	Darke 2014 [8]	Torrisi 2020 [7]; Frati 2015 [10]; Di Fazio 2025 [9]	Forensic/pathological literature describes myocardial injury, fibrosis and related structural abnormalities in AAS-associated deaths.	Provides pathological support for direct myocardial injury, although AAS-specific causal attribution may be limited by polypharmacy and other co-exposures.
Arrhythmogenic substrate	Windfeld-Mathiasen 2025 [6]	Torrisi 2020 [7]; Di Fazio 2025 [9]	The Danish cohort showed increased arrhythmia risk; secondary pathological literature describes fibrosis, hypertrophy and myocardial injury that may facilitate electrical instability.	Clinical and pathological evidence is compatible with an arrhythmogenic substrate, but mechanistic causality is not established.
Sudden cardiac death	Vecchiato 2025 [24]; Vecchiato 2026 [25]	Torrisi 2020 [7]; Di Fazio 2025 [9]	An elevated SCD burden was reported in competitive bodybuilding cohorts; individual AAS exposure was not systematically ascertained.	Supports a contextual SCD signal but cannot establish an AAS-specific causal effect.

Evidence domain	Primary studies	Supporting secondary evidence	Key findings	Interpretation
All-cause mortality	Petersson 2006 [12]; Thiblin 2015 [13]; Windfeld-Mathiasen 2024 [15]	—	Comparative cohorts with individually ascertained AAS exposure consistently showed excess all-cause mortality.	Primary hard clinical endpoint supporting an association between individually ascertained AAS exposure and higher all-cause mortality; causal inference remains limited by observational design.
Natural vs unnatural mortality	Windfeld-Mathiasen 2024 [15]; Darke 2014 [8]	—	Both natural and unnatural death pathways were observed; forensic cases frequently involved additional psychoactive substances.	Suggests that biological toxicity and behavioural/poly-drug pathways may coexist in AAS-associated mortality.
Polypharmacy / stacking	Darke 2014 [8]; Heo 2026 [26]	—	Multiple AAS/performance-enhancing drugs and psychoactive co-exposures were common in forensic/pharmacovigilance evidence; stacking was associated with more serious reported outcomes.	Potential effect modifier and important source of confounding when attributing harm specifically to AAS.
Cardiac function in former users after cessation	Rasmussen 2018 [4]; Abdullah 2024 [5]; Fyksen 2025 [23]	—	Former users showed residual functional differences compared with AAS-naïve controls, while longitudinal follow-up suggested improvement in some measures after discontinuation.	The comparative meta-analysis quantifies residual group differences and should not be interpreted as a common within-person recovery trajectory.

Primary studies are reports retained in the systematic-review evidence set. Supporting secondary evidence comprises reviews or meta-analyses used to contextualize mechanisms and interpretation; these reports are not counted as included primary studies in the PRISMA flow diagram.

AAS, anabolic-androgenic steroids; MI, myocardial infarction; SCD, sudden cardiac death.
