# Supplementary material for: Anabolic–Androgenic Steroid Use, All-Cause Mortality, and Major Cardiovascular Outcomes: A Systematic Review and Meta-Analysis
Source: Med Sci (Basel). 2026 Oct 15;14(6):678. doi: 10.3390/medsci14060678 (PMC13707093; doi:10.3390/medsci14060678)
Supplement: Supplementary file 1 [file medsci-14-00678-s001.zip › Supplementary Table S5.pdf]

Supplementary Table S5. Study-level ROBINS-E risk-of-bias assessment

Judgements apply to the results contributing to the mortality or post-cessation cardiac-function syntheses. ROBINS-E categories are Low risk, Some concerns, High risk, and Very high risk. Overall judgements reflect the highest-impact domain(s), with particular emphasis on residual confounding and selection. These assessments were performed independently by two reviewers with adjudication by a third reviewer when required.

Study / result	Confounding	Exposure measurement	Selection of participants	Post-exposure interventions	Missing data	Outcome measurement	Selection of reported result	Overall
Petersson 2006 – all-cause mortality	High risk	Low risk	High risk	Some concerns	Some concerns	Low risk	Some concerns	High risk
Thiblin 2015 – all-cause mortality	High risk	Low risk	Some concerns	Some concerns	Low risk	Low risk	Low risk	High risk
Windfeld-Mathiasen 2024 – all-cause mortality	High risk	Some concerns	Some concerns	Some concerns	Low risk	Low risk	Low risk	High risk
Rasmussen 2018 – global longitudinal strain	High risk	Some concerns	High risk	Some concerns	Some concerns	Low risk	Some concerns	High risk
Abdullah 2024 – global longitudinal strain / ejection fraction	High risk	Some concerns	High risk	Some concerns	Some concerns	Low risk	Some concerns	High risk
Fyksen 2025 – global longitudinal strain / ejection fraction	High risk	Some concerns	High risk	Some concerns	High risk	Low risk	Some concerns	High risk

Key rationale: objective analytical testing reduced exposure-measurement bias in Petersson and Thiblin. The Danish registry cohort had robust outcome ascertainment but residual confounding and some uncertainty inherent to anti-doping sanction-based exposure classification. Imaging studies were limited by small selected samples, variable cessation ascertainment and timing, and residual confounding from training and co-exposures; the long-term Fyksen follow-up additionally had substantial attrition from the original cohort.
